# Juntos: A Support Program for Families Impacted by Congenital Zika Syndrome in Brazil

**DOI:** 10.9745/GHSP-D-20-00018

**Published:** 2020-12-23

**Authors:** Antony Duttine, Tracey Smythe, Miriam Ribeiro Calheiros de Sa, Silvia Ferrite, Maria Elisabeth Moreira, Hannah Kuper

**Affiliations:** a International Centre for Evidence in Disability, London School of Hygiene & Tropical Medicine, London, United Kingdom.; b Instituto Nacional de Saúde da Mulher, da Criança e do Adolescente Fernandes Figueira, Fiocruz, Rio de Janeiro, Brazil.; c Department of Speech and Hearing Sciences, Federal University of Bahia, Salvador, Brazil.

## Abstract

**Background:**

The 2015–2016 Zika virus outbreak in Brazil was unprecedented and resulted in the birth of more than 3,000 children with congenital Zika syndrome (CZS). These children experience multiple complex health conditions and have limited services to support them and their family’s needs.

**Program Development and Piloting:**

An existing family support program for children with cerebral palsy (Getting to Know Cerebral Palsy) was adapted to the Zika context in Brazil through expert consultation. The program was pilot tested at 2 sites among 6 groups of caregivers (total of 48 families) from August 2017 to June 2018. Group observation and focus group discussions with facilitators and participants at the end of each session informed fast-track learning, which was used to tailor the program for future groups. Fast-track learning—adjusting the intervention in real time based on gathered feedback—was found to be a helpful process to inform and hone the program from its initial concept.

**Program Description:**

The intervention, Juntos, is a facilitated participatory group program for caregivers of children who have CZS. The group sessions are cofacilitated by a parent of a child who has CZS and an allied health professional. The group meets for 10 sessions that last 4 hours. Each session includes an icebreaker, activities, and group discussions. Content covers practical information on caring for a child with a developmental disability including that caused by Zika. Psychosocial support forms an important component, and families are guided from the first week to define and develop their own communities of support. Six pilot groups were successfully run in Rio de Janeiro and Greater Salvador, Bahia. The groups gave positive feedback on acceptability and demand.

**Conclusions:**

The program has the potential to be an important tool for community health and social support services in South America in response to Zika. The program can also be applied to children with neurodevelopmental disabilities other than those caused by the Zika virus, which could be important in ensuring families of children with CZS are less isolated.

## Abstract (precis)

Development of Juntos, a community-based family support program for caregivers of children with congenital Zika syndrome, contained innovative approaches such as using mothers as facilitators, fast-track learning, and de-isolation of families affected by Zika.

## Key Messages

- A community-based family group program for caregivers of children with congenital Zika syndrome (CZS) was developed based on an existing program for families of children with cerebral palsy and pilot tested in Brazil.
- Program managers developing group initiatives should consider fast-track learning approaches to adjust their intervention to make it more useful to participants.
- Clinicians and managers of Zika initiatives should consider that caregivers of children with CZS will likely benefit from the ability to engage and exchange with caregivers of children with other neurodevelopmental disabilities.
- Program managers delivering community interventions targeting caregivers should consider engaging expert mothers as group cofacilitators.
- Integrating emotional support activities into groups that address child development is important, and adds value.

Resumo em português no final do artigo..

## BACKGROUND

The Zika outbreak of 2015–2016 in South America caught the international health community unaware. There had previously been no severe health consequences associated with the virus, despite Zika having been known since the 1940s.^1^ ^,^ ^2^ Zika has now been proven to cause developmental impairments in children^3^ ^,^ ^4^ collectively known as congenital Zika syndrome (CZS).^5^ This syndrome includes microcephaly as the most pronounced and documented symptom, which is linked with severe and multiple impairments. Evidence is emerging that Zika also causes an array of other cognitive and physical impairments that may not be immediately apparent at birth. Microcephaly is likely to be the tip of the iceberg in terms of affected children, as more mild or moderate impairments stemming from in utero Zika infection appear to be far more frequent.^6^ Brazil was the most affected country in the outbreak. As of March 2020, Brazil had 3,559 confirmed cases of CZS with an additional 2,871 cases under investigation (total 6,430 cases).^7^

Although CZS and cerebral palsy are separate conditions, because they have similarities, programs designed for caregivers of children with cerebral palsy could provide a strong foundation to adapt a program for the Zika context in Brazil.^13^ One such program, Getting to Know Cerebral Palsy (GTKCP), was developed by the London School of Hygiene & Tropical Medicine (LSHTM) after a childhood disability survey showed that caregivers of children with cerebral palsy in Bangladesh had very little access to information or support regarding the best way to care for their child and that available services were extremely limited.^14^ GTKCP is a 10-session parent-support program held in the community that aims to improve parents’ knowledge and skills in caring for their child and improve the quality of life of parents and children with developmental disabilities. It is hard to estimate the exact reach of the program, but an online community of practice established in 2014 to support the rollout of GTKCP has 412 members across 72 countries who share knowledge and experiences.^15^ ^,^ ^16^ GTKCP focuses on parents of children aged 2 years and older; a new version, the Early Intervention Program (EIP), was developed for parents of children aged younger than 2 years.^17^ Program material is available from www.ubuntu-hub.org.

Because CZS and cerebral palsy have similarities, programs designed for caregivers of children with cerebral palsy could provide a foundation to adapt a program for the Zika context.

### Needs Analysis

From April to August 2017, we conducted a needs analysis to assess the potential value of a community-based program, based on GTKCP, for caregivers of children with CZS in Brazil. The needs assessment involved: (1) tracking and comparing emerging literature on the clinical presentation of CZS with existing literature on cerebral palsy; (2) conducting a literature review on the needs of caregivers of children with CZS and cerebral palsy in middle-income contexts; (3) meeting with caregivers, specialists, and other local stakeholders in Brazil to identify key gaps, challenges, and needs; and (4) reviewing emerging data from a sister study measuring the social and economic impact of CZS on caregivers. A full description of the needs analysis is available.^8^

We found that providing some services for children with complex multiple impairments at the community level could be crucial to address the unmet needs experienced by families of children with CZS in Brazil and may be more affordable than centralized services (which may be difficult or costly to access). Families of children with CZS, particularly those children with more severe impairments, did not have enough access to specialized health and rehabilitative services and informal support groups, and formalized support for caregivers was also limited. There was some concern raised by clinicians that children with mild to moderate impairments stemming from Zika infection were less likely to attend rehabilitation and that these caregivers were an important group to be targeted. Other researchers have also reported on the additional services required to fully address the care needs of children with CZS and their families.^9^ ^–^ ^12^

Families of children with CZS lacked adequate access to specialized health and rehabilitative services, informal support groups, and formalized caregiver support.

Given the results of the needs analysis that identified the unmet support needs of parents in Brazil and the positive reception of the principle of GTKCP for Brazil among local stakeholders, researchers at the LSHTM who had been involved in GTKCP and EIP felt that adapting GTKCP and EIP for the Zika context and Brazilian culture could be potentially useful. Partnership for the project was established between the LSHTM and 2 Brazilian institutions: the Instituto Nacional de Saúde da Mulher, da Criança e do Adolescente Fernandes Figueira (IFF) in Rio de Janeiro, and the Universidade Federal da Bahia (UFBA) in Salvador.

**FIGURE 1.**
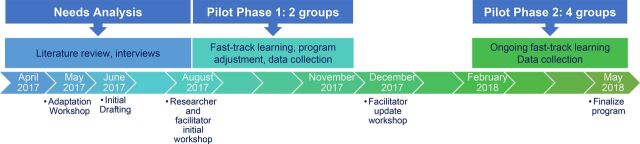
Timeline of Juntos Program Development for Caregivers of Children with Congenital Zika Syndrome, Brazil

This article describes the process of developing and piloting the intervention in Brazil, as well as the final program that was developed (Figure 1). We also reflect on lessons learned as key recommendations from this innovative program may be useful for other global health practitioners designing community-based family group interventions.

## PROGRAM ADAPTATION AND DEVELOPMENT

After conducting the needs analysis, we developed and adapted the program through expert consultation, and then piloted the intervention using a multiphase approach. A protocol was established by the lead project researcher (AD) to measure feasibility of the pilot intervention.^13^

### Ethics Approval

Ethical approval was obtained from the Instituto de Saúde Coletiva/UFBA Ethics Ref 2.369.348, IFF/FIOCRUZ RJ/MS Ethics Ref2.183.547, and LSHTM Ethics Ref 13608. Informed consent was acquired from all participants.

### Initial Adaptation of Program

To support the adaptation, advisory groups were established in Brazil and in the United Kingdom and included a range of specialists, as well as mothers of children with CZS.

The GTKCP and EIP curricula were reviewed by the lead project researcher (AD) with other LSHTM colleagues (TS, HK), Brazilian colleagues (SF, MS), the GTKCP and EIP teams, and other key identified experts (including specialists). The project lead is a physiotherapist with 15 years of programmatic experience, including in qualitative and participatory research and community-based rehabilitation in low- and middle-income countries. During a May 2017 workshop in London, the experts convened to discuss the preliminary findings of the needs assessment and to develop consensus on a first draft outline of the program, an initial timeline, constituency of the facilitators to lead the caregiver group sessions, and participant inclusion criteria.

**FIGURE 2.**
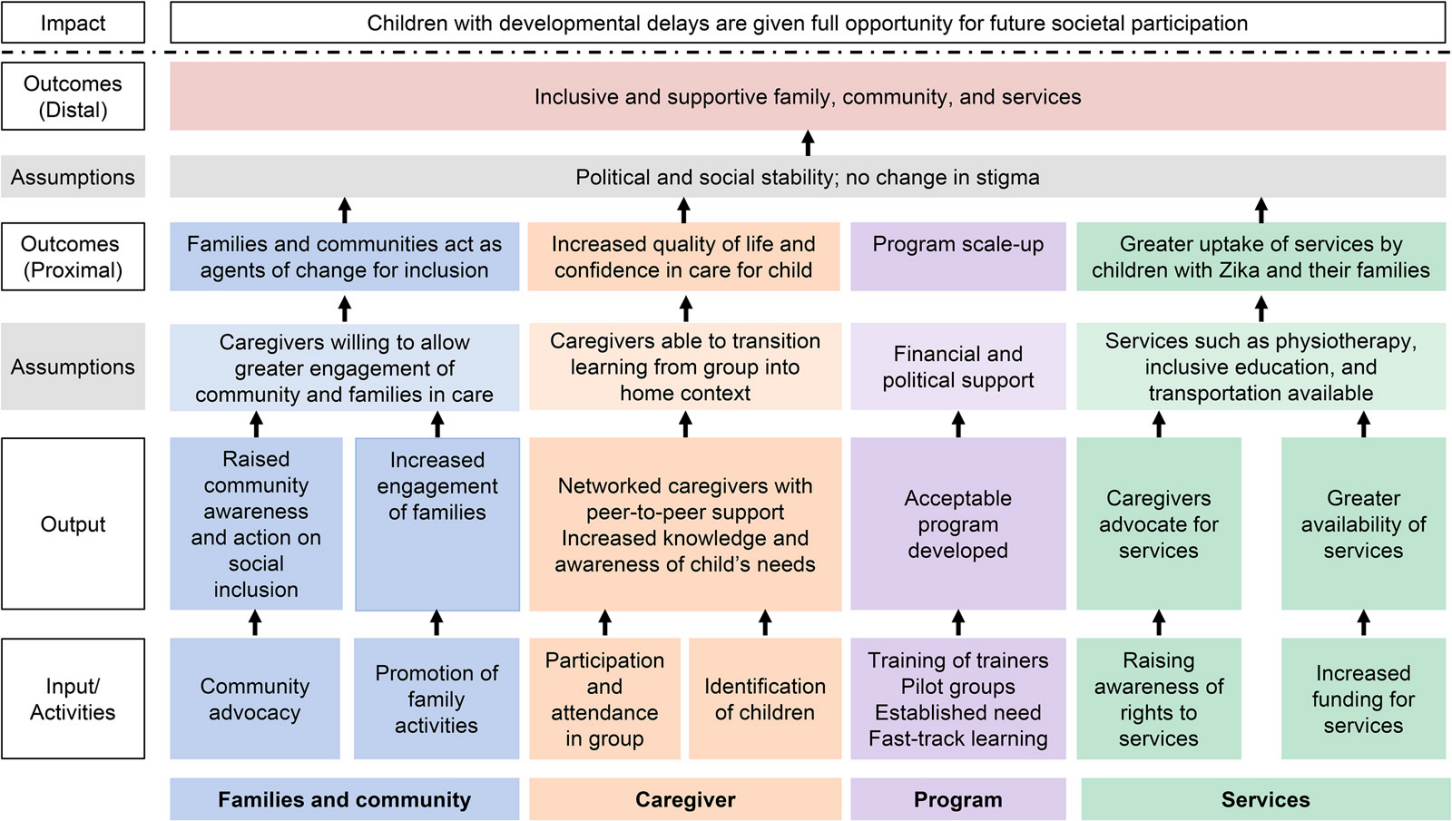
Theory of Change for the Juntos Program for Caregivers of Children with Congenital Zika Syndrome, Brazil

The project group developed a theory of change to describe how the program relates to broader societal participation of children with developmental delays, including CZS, and the pathways that determine the extent to which this intervention may be successful. The theory of change describes what changes are needed and the assumptions underlying the achievement of these changes.^18^ Therefore, the theory of change linked outcomes with activities to explain how and why the desired change was expected to occur and was useful in providing a more comprehensive understanding of steps to improve services to be more inclusive and supportive of family and community. Throughout the program development process, the theory of change was refined to reflect ongoing understanding and research findings (Figure 2).

The theory of change linked outcomes with activities to explain how and why the desired change was expected to occur.

Several areas of adaptation were identified through the emerging literature, clinical experiences of managing children with CZS, development of the theory of change, and by the GTKCP/EIP teams. These areas included recommendations to further strengthen and develop specific approaches to recognize and address caregivers’ psychosocial needs and other clinical issues in children with CZS that were not covered within GTKCP or EIP (e.g., irritability; challenges with breastfeeding or weaning; management of gastrostomy including feeding, low vision, or blindness).

The EIP groups are cofacilitated by an expert mother who has experience caring for a child with cerebral palsy and a rehabilitation professional (e.g., physiotherapist, occupational therapist, or speech and language therapist) who is experienced in working with children who have developmental disabilities. This approach had not been used in GTKCP. A decision was made to pilot test group facilitation by an expert mother combined with a therapist and assess whether this would be effective.

There is a wide range in type and severity of symptoms among children affected by Zika.

It was agreed that program inclusion criteria would be:

- Caregivers of children who have confirmed or suspected CZS but not other types of neurodevelopmental disabilities
- Caregivers of children residing at home and not currently requiring inpatient hospital care
- Caregivers willing to attend the whole program and living within 1 hour of the group meeting location
- Children of any age (although given the nature of the epidemic in Brazil in 2017–2018, they were all aged 3 years and younger)
- Children who may be receiving rehabilitation services to address individual needs

More than 1 caregiver of a child (e.g., mother and father, mother and grandmother) was allowed to attend the group meeting.

From June to August 2017, the project lead researcher (AD) drafted the initial program, adapting the GTKCP and EIP materials with input from expert committee members, project teams in Brazil, and other experts (TS, MS, SF, EM, HK).

## PILOTING

We piloted the approach during 2 phases with 6 different groups and used this information to finalize the program. A future analysis will report the feasibility of the program using qualitative and quantitative data analysis.

### Program Establishment in Brazil

The partners in Brazil (IFF and UFBA) identified a site coordinator (MS and SF) for each of the 2 pilot sites, the states of Rio de Janeiro and Bahia. The site coordinators’ main responsibilities were to manage the logistic components of the pilot groups, including identifying an appropriate location for the groups, recruiting facilitators, recruiting researchers, identifying participants, and liaising with local health providers.

Rio de Janeiro and Greater Salvador, Bahia, were selected as pilot sites because they had a large population of children impacted by Zika. Recife, which the LSHTM team visited during the initial country visit, was not selected because several other intervention projects by other organizations were already taking place and contamination of outcomes was a concern. Three sites within Rio de Janeiro and 3 municipalities of Greater Salvador (Simões Filho, Lauro de Freitas, and Camaçari) were selected because of their proximity to families of children with CZS, availability of an appropriately sized venue, and willingness of the local relevant authorities to accommodate a group.

Facilitators were identified by the site coordinators and approved by the project team lead. A total of 8 local facilitators were selected (4 therapists with experience in pediatrics and CZS and 4 expert mothers). In August 2017, a week-long facilitator training was conducted in Rio de Janeiro and led by a trainer who has taught the GTKCP program extensively. The trainer was international, and we used a translator for the sessions as well as materials in Brazilian Portuguese. The training involved education on facilitating a group, practice sessions with reflective learning and feedback, and opportunities for discussion. The project leads and site coordinators selected 2 pairs of facilitators to lead the first pilot groups based on their performance during the training week.

Two researchers were identified by the site coordinators and approved by the project team lead. All the researchers had a background in psychology, but this was not a prerequisite for the role. The researchers participated in a 2-day training in July 2017 on the research approaches and data collection methods and on the fast-track learning approach that would be used to update and adjust the program content based on weekly feedback that they collected from the groups.

### Pilot Phase 1

In August 2017, the first 2 pilot support groups—1 in Rio de Janeiro and 1 in Greater Salvador—started meeting weekly. The Rio group had 7 families, and the Greater Salvador group had 8. There were 10 sessions for each group with a different topic each week. Researchers used 3 techniques to collect data to inform real-time feedback and fast-track learning about the content and processes of the session. First, researchers directly observed the sessions and noted the session flow, participants’ responses, and behaviors of participants and facilitators. Second, researchers conducted focus group discussions at the end of each of the 10 sessions with participants and (separately) with facilitators to obtain immediate reflections and feedback on the session content. The researchers recorded detailed observation notes about the session and comprehensive notes about focus group discussions that they uploaded to a password-secured Google Drive document for the content developer (TS) to analyze. Third, researchers recorded pertinent comments from participants, facilitators, and site coordinators on images, content, activities, practicalities, and logistics, which were made outside of the sessions. Weekly calls within 48 hours of the session occurred between the researchers and TS, which allowed for further explanation and contextualization. Content issues were recorded and reviewed to update the program in real time and for 4 weeks after the conclusion of phase 1 in November 2017.

### Pilot Phase 2

In December 2017, a 3-day training session provided facilitators and site coordinators with information on the changes to the program content and structure based on fast-track learning in the first pilot phase.

Two additional support groups were established in each pilot setting (4 total), with the primary aim of ascertaining the feasibility of the intervention. These support groups had identical procedures for data collection, real-time feedback, and fast-track learning (February–June 2018). After the delivery of the groups, the intervention was further updated, improved, and finalized using the same processes as before. The 2 groups in Rio had 7 and 9 families, respectively, and the 2 groups in Greater Salvador had 10 and 7 families, respectively.

### Summary

Six groups ran between August 2017 and June 2018 across 2 phases. The children of the caregivers were 25 males and 23 females with an average age of 23 months (standard deviation=9 months) at their first session. Of the families included in all 6 pilot groups, all (n=48) stated the mother as the primary caregiver. The ages of the mothers (n=48) were 15–20 years (3), 21–25 years (17), 26–30 years (5), 31–40 years (18), and 41–50 years (3). Thirty-six mothers reported they were married, 3 divorced, and 9 reported they were single. Only 6 mothers reported being in work, with the most common reason for not being in work being that they cared for their child (n=34).

During the second and third groups in Greater Salvador, held between January and June 2018, several children with non-Zika related developmental disabilities participated in the sessions. This was done for 2 reasons: (1) to increase the number of children participating because the number of children with CZS who met the inclusion criteria was quite low, and (2) to assess whether combining caregivers of children with CZS and those with other neurodevelopmental disabilities would be a positive experience.

We focused primarily on the caregiver and the program, with some interaction with the family, community, and services at the activity and output levels as informed by our theory of change (Figure 2). The proximal outcomes of the program are expected to be (1) increased participant quality of life and confidence in caring for a child with CZS, and (2) an intervention that is feasible to scale up and replicate in other contexts. Core to the theory of change is empowering the caregiver to improve care for their child through developing support networks and increased knowledge and awareness of their child’s needs.

Fast-track learning meant that the intervention was updated and improved as new information was gathered each week about what was working or not. For example, practical or administrative issues, such as organization of transport for participants, were changed and updated in real time each week.

Through fast-track learning, we updated and improved the intervention as new information was gathered each week about what was working or not.

As a result of rapid participant feedback, we made several changes to the program. For example, we changed the title of session 8 (highlighting advocacy and empowerment) to “uniting our voices”; the original title “raising our voices” translated to “shouting out loud.” In a second example, participants felt that the images used in the first 2 pilots, which used images from GTKCP and EIP, did not adequately reflect phenotype, family behavior, and environment in Brazil. Therefore, as participants requested, we included images that reflected their lives to create identification and favor more adherence. A local artist was engaged to draw more culturally appropriate images for the later groups, which were perceived more positively. More representation of fathers in caring roles was also incorporated at this stage.

New innovations in Juntos, which were not in GTCKP or EIP, include information on the Zika virus, strengthened participatory approaches to engage participants with community inclusion and disability rights, and a concerted effort to improve male engagement,^19^ which was successful to a degree (though the female engagement was still much higher). Additional content includes group discussion on gastrostomy (dysphagia was a common problem), creating trousers stuffed with padding to support children in sitting, using an elasticated cloth to rock children who are irritable, and activities to promote understanding of disability rights. In addition, each session includes reflection and discussion on the session and on the past week through an emotional support activity at the end of the session. The facilitators work as a pair together throughout the session; however, the emotional support activity is facilitated by the expert mother. The first 5 sessions include the same activity with facilitated questions:

- How did you find talking about today’s subject?
- Did it raise any emotions or feelings that you did not expect?
- How have you been feeling this week?

The predictability of the questions helps participants to become comfortable with sharing. By week 5, participants have explored much of their thoughts on emotions and feelings, and this then progresses to reflecting on the future.

The feasibility assessment is not detailed in this article and will be described in a future article on the findings.

### Finalization of the Program

Consensus on the final content of the program was reached through 2 workshops (London, United Kingdom, and Rio de Janeiro, Brazil) in May 2018. One group in Greater Salvador was still running. However, feedback that had already been collected from the groups was deemed sufficient to be able to finalize the content. The workshops included the technical advisory committees, study site coordinators, and researchers (psychologists).

## PROGRAM DESCRIPTION

The final program intervention is called Juntos, which means together in Portuguese and Spanish, to emphasize the importance of inclusion and mutual support. Intervention materials comprise a facilitator manual and participant materials, such as photographs, animations, and video footage. An allied health professional and an expert mother cofacilitate groups that meet once a week for 10 sessions. Support and guidance for facilitators is provided by project coordinators via telephone, email, and/or WhatsApp.

**Figure:**
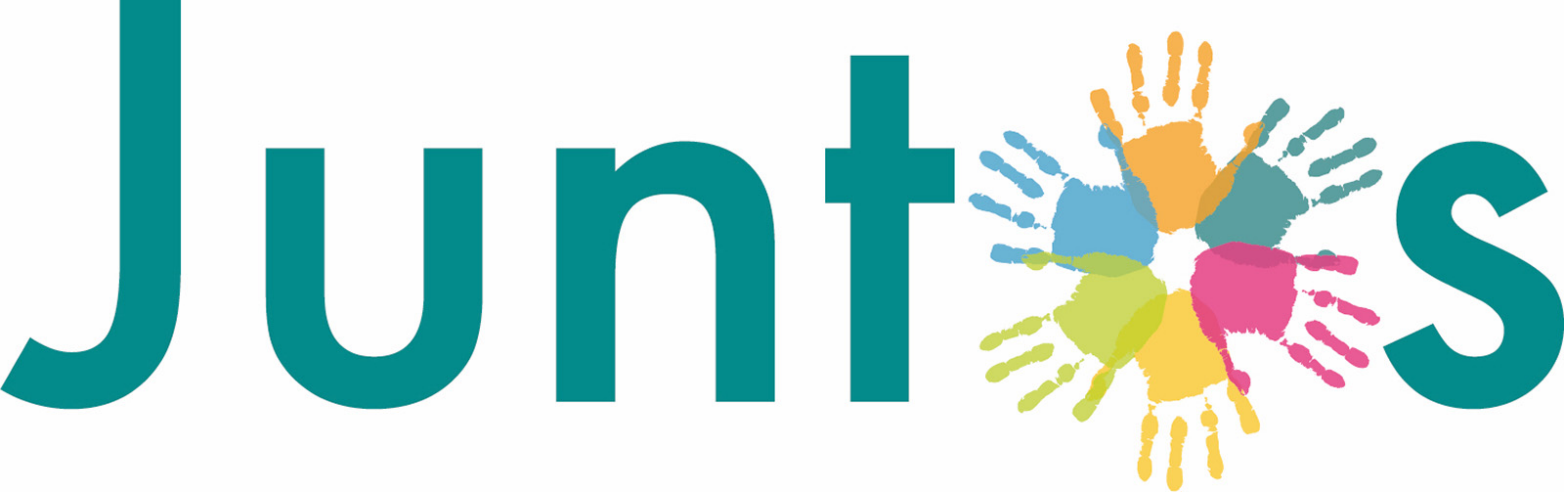
The Juntos logo, which facilitators, psychologists, and site coordinators created to complement the program name’s meaning.

Groups are held at local community facilities, such as health centers, offices of local organizations, or schools, to minimize participants’ travel time and to foster relationships between people who lived relatively near to each other. Nine sessions are only for the caregivers and their children, and 1 session is open for other community members to attend. The children who come are looked after in a separate room or space by volunteers, but they are present for some of the practical aspects whenever relevant. Table 1 describes each Juntos module.

**TABLE 1.** Finalized Module Topics Included in Juntos, A Community-Support Group for Caregivers of Children with Congenital Zika Syndrome in Brazil

Module	**Topics**
1. Introduction	About the program Information about Zika and Congenital Zika Syndrome How to find information Personal stories
2. Our child	Introducing your close family and friends Development milestones for young children Determining your child’s progress Managing irritability and crying
3. Positioning and moving	How to position children who need assistance How to assist children to learn to move
4. Eating and drinking	Feeding challenges Practical skills to address challenges for your child
5. Communication	Importance of communication Practical advice to help your child communicate
6. Play and early stimulation	Importance of play for children to develop and learn Early stimulation Making simple toys Inclusion of play in the family and broader community
7. Everyday activities	How to use everyday activities to help your child develop Managing seizures
8. Uniting our voices	Understand the context of disability rights Education Communicating with your health team Advocating
9. Our community	Who is in your community? Common barriers to inclusion Addressing negative attitudes and exclusion Social activity
10. Next steps	Summing up Planning next steps for yourself and the group

The sessions are participatory and use principles of adult learning theory.^20^ Participants learn by sharing their own experiences and realities about topics that are important to them, which promotes peer support, critical thinking, and mutual problem solving. The groups start with a light-hearted icebreaker to welcome and warm up the conversation and to encourage comfortable interaction. Participants are then guided through a series of activities, open discussions, pair work, explanations, and demonstrations. Tables 2 and 3 provide examples of session content from session 4 and 6, respectively.

**TABLE 2.** Example of Content From Facilitated Group Session 4 on Eating and Drinking from Juntos, A Community-Support Group for Caregivers of Children With Congenital Zika Syndrome in Brazil

Example	Discussion	Aim
**Icebreaker** In pairs: One person tries to give the other a drink of water in different positions (e.g., head leaning back, turned to one side, or flopping forwards)	How easy or difficult is it to swallow in each position? How does it feel to be fed?	To understand a range of issues that your child may experience with eating and drinking
**Discussion** As a large group to share experiences	What is a nutritious or “balanced” diet?	To know what a balanced diet is and how to maximize your child’s nutritional intake and prevent malnutrition
**Activity** Show a banana and a biscuit and other common food	Discuss—Are the items hard or soft? Can they be made into a smooth puree? How?	To learn ways to feed your child safely

**TABLE 3.** Example of Content From Facilitated Group Session 6 on Play and Early Stimulation from Juntos, A Community-Support Group for Caregivers of Children With Congenital Zika Syndrome in Brazil

Example	**Discussion**	**Aim**
**Icebreaker** In groups of 3: each group is given one inexpensive everyday item (e.g., cup, piece of cloth, container, ball) and everyone uses their imagination to transform the object into something else and acts it out	What is play?	To understand how our imagination works with play and how children have an even greater imagination than adults
**Discussion** As a large group to share experiences	What have you found play helps your child to do? Does your child need to play?	To know that play gives children an opportunity to explore, learn about their environment and to use and develop their senses
**Activity** Toy making, such as making bells and rings with ribbons	Discuss—How can you involve short periods of play in your daily activities? How can you involve other members of your family in playing with your child?	To learn ways for play to be fun, and to see how fun can motivate children to move and learn and how other family members can be included

Supportive information was developed for the program that includes short videos on the program and different aspects of care. The individual modules, full manual, and supportive materials are available in English, Portuguese, and Spanish: https://www.ubuntu-hub.org/resources/juntos.

## LESSONS LEARNED

Fast-track learning added value to the intervention development because it allowed inclusion of language, logistics, content, and culturally specific changes in real time. Participants’ feedback during the first pilot phase was utilized to revise the content (for example, providing case studies, images, and videos of fathers undertaking practical tasks), which may have made the overall content more useful for the later groups. The later groups were aware of this process and recognized some of the changes based on early peers’ feedback. In a context of relative distrust and research fatigue,^21^ this process helped to demonstrate how participant feedback was valued and reinforced that the program was genuinely and specifically intended for caregivers, an area that had been largely overlooked in the wider Zika response.^22^ This could be an important point of learning for global health practitioners implementing community-based group programming: bringing together participants, implementers, and researchers to adapt interventions rapidly as feedback is received. In our approach, although not by initial design, the use of psychologists as researchers and observers provided a unique opportunity for nuanced feedback. This was particularly useful for developing and crafting the messaging and discussions on emotional well-being and psychosocial support.

The fast-track learning process demonstrated how participant feedback was valued and reinforced the program’s focus on caregivers, a group that has been overlooked in the wider Zika response.

The integration of a component of caregiver emotional well-being in this group intervention demonstrates a novel approach to including psychosocial support to better promote emotional well-being as an integral part of health work, rather than being seen as a standalone effort. There is no single recognized theory of how participatory groups achieve their health impacts^23^ and few studies evaluate how and why different support networks improve caregiver and child outcomes. Examples in resource-limited settings include self-help groups for people with mental health conditions, which demonstrate positive impacts on both the people with mental health conditions and their caregivers.^24^ Additionally, women's self-help groups have resulted in improved maternal and neonatal survival.^25^ Our integration of a mental health component in Juntos illustrates that groups that address child development can practically integrate emotional support activities. Facilitators reported that they valued having a dedicated space each week to raise issues of emotional well-being. The practical components of the sessions often raised some emotions for a participant, but there would be little time to explore these, so the final section allowed further exploration and discussion between the group. Evaluation of whether such a strategy can work in other settings is necessary, and negative and unanticipated consequences warrant further evaluation in future work. Having an expert mother facilitate these sessions was particularly important and helped form group connections that might not have been possible with an allied health professional alone.

In understanding pathways to change, the role of the expert mother appears to offer crucial encouragement to shared learning between caregivers and contributes to developing an egalitarian atmosphere, expanding care practices beyond traditional rehabilitation models.^26^ Relating this common ground and a sense of belonging through a social support network provides an environment to improve the knowledge and skills of caregivers.^15^ It was critically important that the 2 cofacilitators were equals, each bringing their own experiences to the process and an expertise and insight that the other did not possess. The allied health professionals immediately saw the value in this, and there was no sense of protectionism or defensiveness that they needed to be the lead or expert given their professional training.

The expert mother appears to offer crucial encouragement to shared learning between caregivers and contributes to developing an egalitarian atmosphere.

Groups were held in the local community so that caregivers could build strong local networks. This also increased interest from caregivers of children with developmental disabilities other than CZS and highlights the importance of de-isolating Zika from other causes of neurodevelopmental disability when developing community support programs. Juntos does not replace health care services but rather seeks to complement services by empowering other caregivers to optimize their child’s care and upbringing.

We received positive feedback during the sessions that combined caregivers of children with CZS with caregivers of children with other neurodevelopmental disabilities. There was a recurrent expression of comfort among the caregivers when engaging with other caregivers in similar situations and circumstances that they were not as alone, unique, and isolated as they had perhaps feared. This was also seen in the sessions where non-CZS caregivers engaged and, in fact, there was a value perceived to understand that the challenges being faced were not unique to only caregivers of CZS. This was also reinforced frequently in session 8 of the Rio sessions, where an external speaker came from a local Down’s Syndrome organization to discuss their advocacy approaches; the sessions were always extremely well received by participants. Although the challenges facing children with CZS and their caregivers remain unique and, to a certain degree, still unknown, there may be an important value to ensure that there are also many common issues faced and a shared approach may be both efficient and useful.

## RECOMMENDATIONS

The needs analysis that we undertook at the beginning of the project^8^ as well as more recent literature^27^ ^,^ ^28^ has highlighted overlaps and similarities between cerebral palsy and CZS. We suggest that children with CZS and their caregivers may benefit by integrating and linking with services and programs for children with other neurodevelopmental disabilities. Rehabilitation/therapy services were already doing this to a large extent, and there seems a good scope for other health and social service providers to also ensure service integration. Conversely, newly formed services as a result of the attention to CZS shouldn’t be exclusive to this population group and should seek to include all families and children who may benefit.

By the nature of its design, Juntos can potentially be implemented by a range of stakeholders, from nongovernmental organizations to public community services to primary health settings. This flexibility may mean that there is a stronger opportunity for Juntos to be scaled up. The universal primary health structure in Brazil—the Sistema Único de Saúde—could be an avenue to further explore. We see opportunities for public/private partnerships also. Cost is clearly a major factor in the potential for scale up. Facilitator training can be done in larger groups to reduce costs. In addition, if the facilitator therapists undertake the role as part of their existing work, these costs may be further reduced. However, we do feel that it is important to remunerate parent facilitators for their work and other costs, such as transport and refreshments, to ensure full participation of families.

### Strengths

Strengths of this pilot include the development process being informed by a theory of change and reflective practice and robust methodology that allowed integration of rapid feedback. Real-time feedback and adaption enabled the development of a culture-specific and language-specific intervention, and the program was developed and refined to meet the needs of caregivers of children with CZS in Brazil. Running the program in 2 sites concurrently (Rio de Janeiro and Greater Salvador) was an important methodological choice for achieving better final version program. Brazil is huge and diverse, and although these 2 sites do not cover the breadth of diversity, piloting in more than 1 site and acquiring different feedback added to the strength of the study.

### Limitations

Our study has limitations. We describe the intervention development, but assessment of feasibility and evaluation of replication and scale-up in other countries is now needed. More work is needed on forming a comprehensive facilitator training program, and further development of the intervention to include all children with developmental disabilities is warranted. If Juntos is found to be feasible, robust studies to evaluate the cost-effectiveness of the intervention will be needed.

## CONCLUSIONS

We developed and refined a participatory community-based group intervention to meet the needs of caregivers of children with CZS. Juntos has the potential to be an important resource for community practice. There is scope to expand across Brazil and in other South American countries and to children with other developmental disabilities.

## Notes

**En português**

**Juntos: Um Programa de Apoio às Famílias Afetadas pela Síndrome Congênita do Vírus Zika no Brasil**

**ABSTRATO**

**Histórico:** O surto, sem precedentes, do vírus do Zika em 2015–2016 resultou no nascimento de mais de 3.000 crianças com a Síndrome Congênita do Vírus Zika (SCZ). Essas crianças experenciam múltiplas e complexas condiçöes de saúde com limitado acesso a serviços de apoio tanto para elas quanto para as suas famílias.

**O desenvolvimento de um programa piloto:** um programa existente de apoio às crianças com paralisia cerebral (*Getting to Know Cerebral Palsy* -Conhecendo a Paralisia Cerebral) foi adaptado para o contexto do Zika no Brasil através de uma consultoria especializada. O programa piloto foi testado em dois locais com 6 grupos de cuidadores (um total de 48 famílias) entre agosto de 2017 e junho de 2018. Grupos focais e de observação com facilitadores e participantes receberam avaliações ao final de cada intervenção que foram utilizadas para adequar o programa para grupos futuros, através da metodologia de aprendizagem rápida. Isso permitiu ajustar as intervenções em tempo real, o que provou ser um processo útil para informar e aprimorar o programa desde a sua concepção inicial.

**Descriçäo do programa:** a iniciativa Junto é um programa de facilitaçäo e participaçäo para grupos de cuidadores de crianças com SCZ. São dez encontros com a duraçäo de 4 horas - cada um inclui uma dinâmica inicial de quebra gelo, atividades e discussöes em grupo. O conteúdo cobre informaçöes práticas sobre os cuidados com crianças com problemas de desenvolvimento, incluído aqueles causados pelo Zika. O apoio psicossocial abrange um componente importante no qual as famílias são orientadas desde a primeira semana sobre como definir e desenvolver suporte em suas comunidades. A realizaçäo com seis grupos no Rio de Janeiro e na aérea metropolitana de Salvador ocorreu de forma exitosa e em ambos os locais houve um retorno positivo em termos de aceitaçäo e demanda.

**Conclusões:** o programa tem o potencial de ser uma ferramenta importante para as aéreas de saúde e prestação de serviços sociais na América do Sul em resposta ao vírus do Zika. Além disso, pode ser adaptado para crianças com problemas no neurodesenvolvimento para além daqueles causados pelo Zika, o que por sua vez, pode ser importante para garantir que as famílias de crianças com SCZ sintam-se menos isoladas.

**Aspectos Principais**

- Um programa de orientação comunitária para grupos de família desenvolvido para os cuidadores de crianças com a Síndrome Congênita do vírus Zika (SCZ), baseado em programa anterior focado em crianças com paralisia cerebral, foi testado como uma experiência piloto no Brasil
- Gestores que desenvolvem atividades de grupo devem considerar as abordagens de aprendizagem rápida para adequar as suas intervenções, tornando-as mais úteis para os participantes. Médicos e gestores de iniciativas para o apoio às vítimas do Zika devem atentar para o fato de que cuidadores de crianças com CZS podem se beneficiar da interlocução e troca com cuidadores de crianças com outras problemas de neurodesenvolvimento.
- Gestores de programas focados em intervenções comunitárias devem levar em consideração engajar as mães como um grupo de cofacilitadoras.
- Integrar atividades de apoio emocional em grupos para abordar a questão do desenvolvimento infantil é algo prático importante e agrega valor.

## Notes

**Peer Reviewed**

**Funding:** The project was funded by Wellcome Trust and Department for International Development, Grant Number 206719/Z/17/Z.
